# Cerebral Small Vessel Disease

**DOI:** 10.3390/ijms21249729

**Published:** 2020-12-20

**Authors:** Jakub Litak, Marek Mazurek, Bartłomiej Kulesza, Paweł Szmygin, Joanna Litak, Piotr Kamieniak, Cezary Grochowski

**Affiliations:** 1 Department of Neurosurgery and Pediatric Neurosurgery, Medical University of Lublin, 20-954 Lublin, Poland; marekmazurek@hotmail.com (M.M.); kuleszabartek88@gmail.com (B.K.); pawelszmygin@wp.pl (P.S.); p.kamieniak@onet.pl (P.K.); 2 Department of Immunology, Medical University of Lublin, 20-093 Lublin, Poland; 3 St. John’s Cancer Center in Lublin, 20-090 Lublin, Poland; litak.joanna@gmail.com; 4 Department of Anatomy, Medical University of Lublin, 20-090 Lublin, Poland; cezary.grochowski@o2.pl; 5 Laboratory of Virtual Man, Department of Anatomy, Medical University of Lublin, 20-090 Lublin, Poland

**Keywords:** CSVD, CMB, cerebral microbleeds, cerebral small vessel disease

## Abstract

Cerebral small vessel disease (CSVD) represents a cluster of various vascular disorders with different pathological backgrounds. The advanced vasculature net of cerebral vessels, including small arteries, capillaries, arterioles and venules, is usually affected. Processes of oxidation underlie the pathology of CSVD, promoting the degenerative status of the epithelial layer. There are several classifications of cerebral small vessel diseases; some of them include diseases such as Binswanger’s disease, leukoaraiosis, cerebral microbleeds (CMBs) and lacunar strokes. This paper presents the characteristics of CSVD and the impact of the current knowledge of this topic on the diagnosis and treatment of patients.

## 1. Introduction

Cerebral small vessel disease (CSVD) represents a cluster of pathologies with a heterogeneous etiology and a pathomechanism affecting elements of the brain vascular system such as small arteries, capillaries, arterioles and venules. Histopathologic studies demonstrate reduced lumens in affected vessels and also demonstrate the thickening of walls, which impedes perfusion and transmural gas transfer [1]. The disease accounts for 20–30% of cases of ischemic stroke [2,3] and cerebral hemorrhage [4,5]. Moreover, CSVD has been shown to worsen functional outcomes after supra [6] and infratentorial [7] ischemic stroke because it disrupts the reorganization of brain networks that is essential for post-stroke recovery. Certain fluid biomarkers have been identified to correlate with CSVD. Some studies present elevated levels of Low Molecular Weight Neurofilament Protein (NF-L), tissue inhibitor of metalloproteinase-1, metalloproteinase-9 and metalloproteinase-2 in CSVD patients [8]. Imaging examination has revealed a direct relationship between Alzheimer’s Disease occurrence and certain identified cerebral vascular diseases, principally CSVD.

CSVD can be classified according to varied pathological, radiologic and clinical criteria. Most commonly, two types are identified: amyloid and non-amyloid related (Table 1). CSVD has been recognized as a dynamic condition of the whole brain and as having a diffuse nature, and systems for the visual scoring of MRI images have been introduced to assess the total load of the disease [9,10]. The neuroimaging features are white matter hyperintensities (WMH), enlarged perivascular spaces (EPVS), lacunae, subcortical infarcts, microbleeds and brain atrophy. Some researchers include individual disease entities in this group, such as Binswanger’s disease, leukoaraiosis, cerebral microbleeds (CMBs) and lacunar strokes.

The following review presents the most up-to-date findings on the conditions belonging to the group of CSVD. The first section covers the pathology of particular types of CSVD, and the second section explains their clinical manifestations and significance.

## 2. Classification of Cerebral Small Vessel Disease

CSVD can be divided into six groups:

○ Type I: arteriosclerosis/age-related CSVD;
○ Type II: amyloid-related CSVD;
○ Type III: genetic CSVD distinct from amyloid angiopathy;
○ Type IV: inflammatory/immunologically mediated CSVD;
○ Type V: venous collagenosis;
○ Type VI: other CSVD.

Of these, the most common in the population is Type I, which is associated with arteriolosclerosis, and Type II, which is caused by amyloid angiopathy. Table 2 shows a comparison of their characteristics. The etiology of CVSD is complex and includes many mechanisms depending on the type of CSVD. In arteriosclerosis/age-related CSVD, the density of smooth muscles in the tunica media is decreased, and fibro-hyaline deposits narrow the lumen of the arterioles. This form is closely associated with systemic angiopathies and has similar risk factors; i.e., aging diabetes and hypertension [11]. Amyloid-related CSVD is characterized by the gradual accumulation of amyloid in the walls of arterioles, especially the leptomeningeal and cortical walls, which become fragmented, and blood extravasation ensues [12]. This angiopathy is commonly observed in Alzheimer’s disease and Down’s syndrome [13]. The prevalence of amyloid-related CSVD increases with age. It can result in large lobar hemorrhages [14] as well as cerebral microbleeds [15]. In the group of genetic CSVD distinct from amyloid angiopathy, the most well-described examples are cerebral autosomal dominant arteriopathy with subcortical ischemic stroke and leukoencephalopathy (CADASIL) and Fabry’s disease. Genetic mutations determine the formation of endothelial deposits, such as glycosphingolipid GB3 in Fabry’s disease, causing stroke in 24% of patients or even more commonly small vessel infarction [16]. Numerous genetic studies have focused on elucidating the hereditary background of familial CSVD. Highly penetrant mutations involve the following genes: NOTCH3, HTRA1, TREX1, GLA, COL4A2 and FOXC1 [17].

Inflammatory CVSD is often a component of systemic diseases. The common feature of these diseases is the presence of inflammatory cells in the vascular walls, known as vasculitis. This phenomenon concerns, among others, IgA vasculitis, eosinophilic granulomatosis with polyangiitis, granulomatosis with polyangiitis, cryoglobulinemic vasculitis, cutaneous leukocytoclastic and angiitismicroscopic polyangiitis. The most prominent type of inflammatory/immunologically mediated CSVD is primary angiitis of the central nervous system (PACNS), a rare vasculitis triggered by infection. Cytomegalovirus, Epstein–Barr, varicella-zoster virus, HIV, mycoplasma and chlamydia are the agents responsible for the initiation of Th1, tumor necrosis factor (TNF) and interleukin (IL)-6-mediated inflammatory/immunological response [18,19]. T-lymphocytes and macrophages infiltrate vessel walls, leading to the occlusion of the lumen. The diagnosis of PACNS is delivered through blood samples, CFS analysis and conventional angiography, which reveals multiple beadings or the segmental narrowing of the vessels [20].

Venous collagenosis is the name proposed by Moody et al. to describe the thickening of periventricular veins [21]. The consequence of the obstruction of the venules is reduced perfusion pressure and disordered venous outflow. The material causing the narrowing of the vessel’s lumen is mainly an excess of collagen. When chronic, it can lead to local low-grade edema and demyelination, which manifests as white matter hyperintensities. The last group of CVSD includes, inter alia, post-radiation lesions, which are a side effect of brain radiation therapy. Their onset is usually delayed; they may occur months or years after treatment. The effect of radiotherapy is fibrinoid necrosis and the deposition of hyaline material in small vessels present mainly in white matter. This causes the thinning of the vessel wall and the narrowing of its lumen, disturbing the flow. The occurrence of post-radiation CVSD is therefore associated with diffuse leukoencephalopathy with accompanying demyelination. Additionally, several studies have reported inflammatory cells in brain parenchyma and perivascular spaces [22,23], and more recently, the transmural infiltration of vascular structures could be detected in patients treated with Gamma Knife radiosurgery for brain metastases [24].

## 3. Etiology of Cerebral Small Vessel Disease

The deposition of amyloid-beta in the cerebral vessels (cerebral amyloid angiopathy (CAA)) is a common finding in elderly people, a major cause of spontaneous intracerebral hemorrhage (ICH) and an important contributor to age-related mental decline [25]. The accumulation of eosinophilic hyaline material along the basement membranes is accompanied by progressive loss of the smooth muscle layer in the media of arterioles [26]. This results in the formation of microaneurysms and a temporary blockage of the vessel lumen [27]. However, the exact etiology of these lesions remains unknown. Some authors associate amyloid deposition in CAA with its production in smooth muscle cells after earlier damage [28,29]. The neuronal origin of β amyloid, which in the next stage would be transported into the blood along the fluid spaces around the cortical and meningeal arteries, has also been suggested [30,31]. Other theories concern the damage to the blood–brain barrier, which determines the penetration of its molecules [32,33].

The pathological hallmarks of CAA, first described in 1954 by Stefanos Pantelakis, have since been confirmed by more recent studies: the involvement of arterioles of the meninges and cerebral cortex, favoring posterior regions of the brain (occipital lobes), the sparing of white matter vasculature, association with age and dementia, a lack of association with hypertension and atherosclerosis (unlike other CSVD) and independence from systemic amyloidosis [34]. Cerebral microvessels are crucial for the drainage of interstitial fluid of the brain; thus, the accumulation of amyloid-beta is an early sign of clearance failure and has pending hemorrhagic or ischemic consequences [35]. In an autopsy, the condition can be detected in 20–40% of the non-demented and 50–60% of the demented elderly population [36].

Although highly prevalent in patients affected with Alzheimer’s disease (found post-mortem in 85–95% of patients), it is rarely diagnosed during the patient’s lifetime [37]. In MRI, biomarkers of CAA are lobar ICH, lobar microbleeds, cortical superficial siderosis, white matter hyperintensities (periventricular, posterior–predominant), enlarged perivascular spaces in the cerebral white matter and cortical microinfarcts. CAA lesions primarily affect the cortical vessels and those that supply the meninges. This determines the location of the markers detected in imaging tests. It has been shown that, in the case of changes caused by amyloid etiology, microbleeds, which are a sign of CSVD, are usually found in the cerebral and cerebellar lobes, including their cortical and subcortical areas [8]. Localization in the occipital lobe is particularly frequent [2]. It is also worth mentioning that the presence of such changes may also be genetically determined. An analysis of the genotype of apolipoprotein E showed its ε4 isoform to be associated with a greater prevalence of microbleeds. At the same time, it results in a greater ratio of β-amyloid 40 to β-amyloid 42 [38]. CAA has been classified into two pathological types: CAA type 1, characterized by amyloid in cortical capillaries; and CAA type 2, where amyloid deposits are found in leptomeningeal and cortical arteries, but not capillaries [29].

Another key factor in the etiology of cerebral small vessel disease is hypertensive angiopathy—the umbrella term for a spectrum of sporadic non-amyloid small vessel pathologies associated with age, hypertension, diabetes mellitus and other vascular risk factors. Pathologically, it is characterized by the narrowing of the vessel lumen resulting from the collagenous hypertrophy of the vascular wall and exudation of serum proteins [1]. This type of microangiopathy predominantly affects the small perforating arteries of the deep grey nuclei and deep white matter [39]. As a consequence, the bleedings caused by this condition occurs in deep-brain regions (e.g., the basal ganglia, thalamus and brain stem). Distinguishing CAA from hypertensive angiopathy may have clinical implications (which is relevant for treatment decisions concerning antithrombotic use), as CAA-related lobar ICH carries a considerably higher risk of recurrence [40].

Recent studies have proven that oxidative stress is also a major factor influencing different types of CSVD. Oxidative stress is caused by disturbances of the homeostasis between oxidation and antioxidation processes. An imbalance occurs when free radicals increase or antioxidation processes become inefficient [41,42]. Molecular oxygen undoubtedly plays key roles in the biology of every cell, thus affecting tissues and systems and consequently the entire organism; it is necessary for proper functioning and life [43,44,45]. Although oxygen is important as a life-determinant and is also involved in signal transduction, the regulation of gene transcription and the control of other cellular activities, it also has a detrimental effect on biomolecules in the form of reactive oxygen species (ROS) and free radicals. The unfavorable effect of oxygen is due to its monovalent reductive status, which is directly responsible for ROS production [43,44,46]. Oxygen is an irreplaceable entity for all living organisms, although its presence in excess has harmful effects. Therefore, it is required that the consumption and uptake of oxygen be maintained under a high level of control and that the levels are checked by a complex cell system [46,47,48,49]. Uncontrolled redox reactions generate ROS, such as hydroxyl radicals (•OH), superoxide anions (•O_2_^−^), peroxyl radicals (ROO•), hydrogen peroxide (H_2_O_2_) and nitric oxide (NO•). Hydrogen peroxide (H_2_O_2_) and superoxide anion (O_2_^−^) constrict vessels, reducing blood flow [50,51,52,53]. Oxygen radicals activate inflammatory processes and the formation of oxidized low-density lipoprotein (LDL), affecting the vascular endothelial part of the vessel walls [54,55,56]. Oxidative stress is an indisputable factor contributing to vascular damage and loss of function. Nitric oxide is another important mediator that could be a target of the destructive influence ROS and carries out a regulatory function on vascular smooth muscle cells. It controls many processes such as proliferation and relaxation, vascular tone intensity, hemodynamics and angiogenesis [57,58]. Oxidative stress and ROS are commonly known as crucial factors in the etiology of CSVD. The endothelium, as a significant structure of the vessel architecture, regulates wall tone and maintains adequate perfusion. Many studies have revealed that the endothelium is the main target of inadequate oxidation, accelerating the degenerative effects on CNS blood flow in CSVD patients. Monovalent reactive forms of free radicals are promoted by arterial hypertension, the oxidation of low-density lipoproteins (oxLDL), diabetes mellitus, a high level of homocysteine, general infections and cigarette smoking. Consequently, excessive ROS formation underlies the pathology of cerebral small vessel disease [59].

## 4. Detection of Cerebral Small Vessel Disease

The diagnosis of cerebral small vessel disease is based on the detection of neuroimaging markers occurring in the course of its development. They include a number of characteristic lesions that can be observed in imaging tests. This group includes cerebral atrophy, leukoaraiosis, white matter hyperintensities and cerebral microbleeds.

### 4.1. Cerebral Atrophy

Cerebral atrophy is a kind of condition in which neurons and the connections between them are lost. It causes decreases in brain volume [60,61]. The consequences of this condition manifest in cognitive and neurological problems. Atrophy can be generalized or focal. Focal cerebral atrophy and the corresponding damage affect a particular area of the brain tissue. This type of atrophy can manifest in the corresponding functional impairment of the concerned area of the brain [62,63]. Decreases of brain volume can usually be identified by computed tomography (CT) and magnetic resonance imaging (MRI). Radiological examination may show changes in the brain tissue that are closely related to cerebral atrophy. CT and MRI are equally able to demonstrate cortical atrophy, but MRI is more sensitive to the detection of some types of atrophy, such as focal atrophic changes in the nuclei [64,65,66]. A prospective follow-up study published by Nitkunan et al. showed that brain tissue volume is decreased in patients with cerebral small vessel disease with respect to normal aging subjects. Additionally, this atrophy was associated with cognition decline in 1-year follow-up [67]. Leukoaraiosis research works have proven that decreased brain tissue volume is associated with and facilitates cognitive decline. Brain atrophy due to cerebral small vessel disease is independently related to longitudinal cognitive decline [64]. The size of the white matter located in periventricular and subcortical brain tissue and the number of lacunar infarcts have been associated with the severity of brain atrophy in MRI examination [65,68,69].

Some degree of cerebral atrophy occurs naturally with age. This also applies to many pathological conditions, such as epilepsy, traumatic brain injuries, strokes, multiple sclerosis, Huntington’s disease and cerebral palsy [70,71,72]. An association has been shown between cerebral cortex atrophy and drug and alcohol toxicity, as well as Alzheimer’s disease (AD) [73,74]. A large number of studies have confirmed that cerebral atrophy is the most significant morphological characteristic of AD [75,76,77,78].

### 4.2. Leukoaraiosis and White Matter Hyperintensities

The term leukoaraiosis was introduced in 1987 by Hachinski, Potter and Merskey to describe bilateral periventricular hypodense areas of white matter seen in CT scans, mostly in the elderly population [79]. It roughly corresponds with white matter hyperintensities (WMH), defined as disseminated regions of white matter changes that are hyperintense in T2-weighted findings and FLAIR in MRI images, predominantly around the ventricles and subcortically. Histologically, the atrophy of axons, as well as a decreased quantity of myelin, is observed. This could be the result of an insufficient blood supply to the deep portions of white matter due to vascular pathology [80,81,82]. Makedonov et al. report that the perfusion of white matter hyperintensities (WMHs), as assessed with SPECT and MRI, is lower than the perfusion of normal-appearing white matter [83]. Other researchers point to an impairment of lymphatic drainage as the suspected mechanism rather than an infarction, because no foamy macrophages are present [84]. In patients with beta-amyloid deposits in the basement membranes of arterioles, the interstitial fluid cannot be sufficiently reabsorbed [85]. White matter hyperintensities can be found in 20% of adults in their sixties and in up to 94% in the population of octogenarians [86,87]. They are a common finding in asymptomatic patients; however, the prevalence is higher in the population affected by AD [88,89]. Likewise, patients with cardiovascular risk factors and symptomatic cerebrovascular disease are more likely to develop WMHs [90]. This has clinical implications, as shown in the Perindopril Protection Against Recurrent Stroke Study, where the WMH volume was successfully reduced after 36 months of treatment with ACE inhibitor [91]. Moreover, in a meta-analysis of nine previous studies, Debette and Markus confirmed a significant association of white matter hyperintensities with incident stroke [92]. Furthermore, recent studies have demonstrated the clinical significance of WMH with regard to bladder dysfunction as well as gait and balance disorders [93].

### 4.3. Lacunar Strokes

Lacunar strokes (LSs) of the cerebrum result from the occlusion of small perforating arteries. By definition, the diameter of a lacunar stroke lesion is less than 20 mm on the axial plane [94]. They can be classified by their shape, as tubular (resulting from the occlusion of larger perforating vessels and confluence of lesions) or oval, and by their size, which can be as large as 15–20 mm and as small as 0–14 mm [95]. They comprise around 20% of ischemic strokes [96]. The clinical lacunar syndromes are pure motor stroke, pure sensory stroke, mixed sensorimotor stroke, ataxic hemiparesis and dysarthria/clumsy hand. Very often silent, they are found in 20–50% of elderly people [97]. Hypertension and diabetes mellitus have been established as important risk factors [98,99]. Family history data analysis suggests a hereditary predisposition for lacunar stroke [100]. Large artery abnormalities are often observed in LS, and the possibility of artery-to-artery embolism has been indicated, as shown in a study associating calcifications in the carotid siphon and silent LS [101]. Moreover, CSVD and its sequelae LS are observed to co-exist with abnormalities in small vessels of other organs, including kidneys and retina [102,103]. An important pathological mechanism is endothelial dysfunction leading to the vasoconstriction, inflammation and proliferation of the affected vessels. The circulating markers of endothelial activation, namely intercellular adhesion molecule-1 (ICAM) and thrombomodulin, are elevated in patients with LS as compared with age-matched controls [104]. Furthermore, the LS areas show increased brain–blood barrier permeability, which appears as white matter hyperintensities on MRIs. Lacunar stroke is associated with a lower rate of motor disability and urinary incontinence than stroke due to large-vessel occlusion in anterior or posterior cerebral circulation or hemorrhagic stroke. Similarly, depression is more common in survivors of large-vessel disease (52%) than in patients affected by a lacunar stroke [105]. However, intellectual disability is a serious consequence of this type of stroke, as it is often a manifestation of an underlying diffused condition of cerebral vessels. In total, 11–23% of patients with lacunar stroke will develop dementia [106,107], and the risk increases with recurrent lacunar events [108].

### 4.4. Cerebral Microbleeds

Another manifestation of CSVD is cerebral microbleeds (CMBs). CMBs present as small, oval or round hypointensive lesions in T2 sequences of the brain in MRI [109]. In neuropathological terms, they are perivascular deposits of blood degradation products contained in macrophages. The cause of their formation is blood extravasation as a result of a degenerative status in small vessels and the subsequent decomposition of hemoglobin released from erythrocytes [110,111]. They can be present in several medical conditions as well as disease-free people. CMBs have been shown to occur in 23.5% of the healthy elderly population [112]. An increase in their prevalence occurs, among others, in the case of intracerebral hemorrhage, where they are present in 47–80% of patients. With ischemic stroke, this value ranges from 18 to 71%, while this range for patients with cognitive impairment and dementia stretches from 17 to 46% [113].

The occurrence of microbleeding is influenced by many factors. Microbleeds are present in 17.8% of people aged 60–69 and as much as 38.3% of patients over 80 [114,115], in which their population prevalence oscillates at around 5% [116]. Recent studies have proven the causative role of smoking, an unhealthy diet, arterial hypertension and atrial fibrillation in cerebral microbleeding [117,118,119,120]. On the other hand, it has been shown that diabetes is correlated negatively with cerebral microbleed lesions [121]. The genesis of cerebral microbleeds (CMBs) is very complex, because several pathological processes often take part in their formation. It is now believed that the most important aspect is angiopathy in the course of hypertension and amyloid angiopathy [10,11,12,13].

The presence of CMBs has been proven to significantly affect brain tissue, causing an impairment of its function. The research conducted by Cianhetti et al. suggests that there is a temporary loss of neuronal and astrocyte function in areas adjacent to a CMB. A consequence of these changes may be the occurrence of neurological focal episodes (TFNEs), often reminiscent of TIA or seizures [101,122]. Several observations also indicate the irreversible destruction of brain tissue in the vicinity of a CMB [123,124]. The Rotterdam Scan Study revealed, in a group of people with microbleeding, the condition of seemingly normal white matter of the brain that showed that the appearance of a CMB reduced white matter integrity within its microstructure [124]. The occurrence of microbleeding is not indifferent to the system; several studies show the relationship between its prevalence and the presence of various medical states. A common symptom in people affected by CMBs is cognitive impairment. In an observation carried out in Japan in 518 healthy people without neurological disorders, MRI gradient-echo and mini mental state examination (MMSE) were performed. In the next stage of observation, as part of the Kashima Scan Study, researchers focused on the relationship between the severity of disorders and the exact location of deep and sub-tentacle CMBs [125]. Similar observations were also conducted by Chung et al. as part of the I-Lan Longitudinal Aging Study, which examined 959 patients for abnormalities in specific cognitive domains such as verbal memory, visuospatial functions, language or verbal executive functions. The results showed that the tested domain disorders related primarily to patients with lobar CMBs. The largest relationship existed in the case of visual–spatial deficits of executive functions. However, no similar correlations were observed in individuals with microbleeding localized in the deep and sub-tentative areas of the brain tissue [126]. Such a distribution of lesions indicates the possible role of amyloid angiopathy in the genesis of these disorders. The pathogenesis of the impact of the presence of CMBs on the occurrence of cognitive impairment is not fully understood. Some researchers assume that micro-bleeding disturbs the signal flow between the basal ganglia and frontal lobes [127]. Studies using diffusion MRI have revealed the destruction of the white matter microstructure present in gently expressed cognitive impairment in patients with CSVD. This mainly concerned the anterior part of the major commissure and the outer and inner capsule, while in patients without the coexistence of CVSD, the destruction was limited only to the stabbing path along the hippocampus [128]. However, the cause of the described phenomenon has not been established yet. CMBs are very common in medical conditions involving dementia. They can be found in 29% of patients with Alzheimer’s disease and in 85% of people with vascular dementia [129,130,131,132,133,134,135].

The presence of CMBs could be important for the occurrence of strokes. Among patients admitted after an ischemic stroke, CMBs are found in 34% of cases, and in people after non-traumatic intracerebral hemorrhage, this value is 60% [123]. The frequency increases with relapses of similar episodes. For patients with their first ischemic stroke, the prevalence is 23%, while in subsequent episodes, this value increases to 44%. For hemorrhagic stroke, these figures are 52% for patients with the first episode and as much as 83% for relapses [123]. This is confirmed by a literature analysis performed by Wilson et al. The presence of CMBs can be used in the assessment of the outcome in stroke patients. A lobar location of a CMB causes a greater risk of death from stroke, while other locations result in a greater burden of cardiovascular mortality [136]. The authors showed that the frequency of a recurrent ischemic incident in the CMB group oscillated around 9%, while in people without similar brain changes, this percentage was equal to 5.6% [137]. Observations carried out as part of the Rotterdam Study have shown that the occurrence of multiple CMBs correlates with a higher risk ratio of all stroke types. In addition, a lobar location of CMBs, characteristic for CAA, was connected with a significantly higher risk ratio of intracerebral bleeding, while CMBs in other regions were associated with the incidence of both hemorrhagic and ischemic stroke [138]. It is also worth noting that CMBs were rarer in patients with stroke with a thrombotic etiology with atherosclerotic pathogenesis and in cardiovascular mechanisms [139,140].

## 5. The Significance of Cerebral Small Vessel Disease

### Cognitive Impairment, Dementia and Alzheimer’s Disease

Alzheimer’s disease (AD) is a type of progressive neurodegenerative disease of the CNS that mainly affects elderly people; over 80% of AD patients are over 75 years old [141,142]. In the US, it is estimated that by 2030, every fifth person will be over 65 years of age [143]. The aging of the population is closely related to the occurrence of dementia. The most common cause of dementia is Alzheimer’s disease (AD) [143,144,145]. Despite the high prevalence of the disease in the population, its etiology is still not fully understood. Many authors have drawn attention to the potential role of vascular changes in its pathogenesis. Studies suggest that dementia, particularly in AD, is associated with toatherosclerosis and arteriolosclerosis [146]. Several observations confirm that atherosclerosis in the circle of Willis also causes dementia and dementia from AD [147,148]. Furthermore, atherosclerosis within the small vessels of the brain is independently associated with a higher likelihood of dementia from AD [149]. Vascular cognitive impairment is closely related to cerebral small vessel disease (CSVD), including small subcortical infarcts, lacunae, white matter hyperintensities (WMH), enlarged perivascular spaces (EPVS), microbleeds and brain atrophy [150].

Strokes affecting all types of vessels, from microscopic to large vessels caused by a cerebral infarct, are established risk factors for dementia and cognitive impairment, which has been confirmed in pathological studies [151]. As mentioned earlier, lacunar strokes are of great importance in the etiology of cognitive disorders. Statistics show that as many as 11–23% of patients with lacunar stroke will develop dementia [106,107]. Moreover, the risk is even greater with recurrent episodes [108]. The typical pattern of cognitive deterioration in these patients is impairment in attention and executive function with the preservation of memory [148,152]. The cognitive domain most affected by lesions in the subcortical gray and white matter, as seen in lacunar strokes, is information-processing speed [153,154].

Brain atrophy, especially total WMH and GM volumes, was shown to have the strongest relationship with cognitive dysfunction in a 3-year follow-up. Additionally, the hippocampal volume also has a strong association with global cognitive function and memory domain [150,151]. In AD and CSVD, brain atrophy symmetrically reduces specific gray or white matter volumes, enlarges superficial sulci and increases ventricular volumes. Characteristic manifestations are atrophy changes in the hippocampus on imaging examinations [62,78,155]. Additionally, it has been found that the acceleration of atrophy located in the hippocampus, among patients with mild cognitive impairment, enhances the progress to clinical Alzheimer’s disease, and the progression of AD is strongly related with regional measures of hippocampal atrophy [60,64,78]. Studies show that cerebral atrophy connects both Alzheimer’s disease and CSVD. The relationship between AD and CSVD still has not been completely explained. Determining the exact role of cerebral atrophy in CSVD and AD has become a challenging matter that requires more attention and further observations.

In a post-mortem study of the brain, researchers described changes characteristic of CMB in patients with advanced dementia, which is the main symptom of Alzheimer’s disease [156]. These changes are related to the pathogenesis of CSVD, i.e., the rupture of small arteries or arterioles, disruption of the blood–brain barrier and strokes [157,158]. As mentioned above, these effects can be found in 29% of patients with Alzheimer’s disease and in 85% of people with vascular dementia [129,130,131,132,133,134,135]. Cerebral microbleeding is also a predictor of the occurrence and severity of cognitive impairment in neurodegenerative disorders [131]. Among people with Alzheimer’s disease, a high rate of mortality was observed in the case of the coexistence of microbleeding [128]. Additionally, in the case of Parkinson’s disease, the presence of CMB significantly increases the risk of developing cognitive impairment [135].

White matter hyperintensities—another manifestation of CSVD—are also important in the etiology of cognitive disorders. Observations have shown that this neuroimaging marker is more common in patients with Alzheimer’s disease [88,89]. In cases of dominant AD mutations, WMHs are present as early as 20 years before the onset of cognitive symptoms [85]. WMHs have been linked with vascular dementia and Lewy body dementia [159]. The information processing speed and executive function of cognitive performance seem to be particularly affected in patients with WMHs [160].

## 6. Binswanger’s Disease

One of the pathologies classified as cerebral small vessel disease is Binswanger’s disease (BD). It is a kind of cerebral small vessel disease (CSVD) associated with damage mainly in the white matter. There are some synonyms for the disease, such as subcortical arteriosclerotic encephalopathy, encephalitis subcorticalis chronica progressiva and subcortical dementia [61,62]. This disease was described in Switzerland by Otto Binswanger in 1894. Six years later, Alois Alzheimer first used the term “Binswanger’s disease” [161].) Binswanger’s disease is a form of vascular cognitive impairment (VCI) that is related to the injury of the small vessels of the brain. This special form of injury is characterized by extensive white matter hyperintensities with some kind of gradual subcortical ischemia. Stroke and mental disorder are the first symptoms of BD. This sign usually starts between 55–65 years old, especially after 60 years old. A patient history reveals past episodes of “mini-strokes” or transient ischemic attacks. On physical examination, there are usually upper motor signs, asymmetric hyperreflexia and mild parkinsonism. Symptoms are always steadily progressive [162,163]. Arterial hypertension and other vascular risk factors, including diabetes, pre-diabetes, smoking, hyperlipidemia, sleep apnea and atrial fibrillation are also present [67,164] Binswanger’s disease, as with other cerebral small vessel diseases, is associated with Alzheimer’s disease. There are a large number of risk factors for BD and AD, such as older age, hypertension, atrial fibrillation, diabetes, smoking, sleep apnea and hyperlipidemia, drinking alcohol and obesity [165,166]. However, DB seems to be a potential risk factor for Alzheimer’s disease [167]. The biomolecular relationship between BD and Alzheimer’s disease requires further investigation.

## 7. CADASIL

Cerebral autosomal dominant arteriopathy with subcortical infarcts and leukoencephalopathy (CADASIL) is the most common familial cerebral small vessel disease and strongly correlates with ischemic stroke and dementia. The condition is associated with the degeneration of smooth muscles and stenosis of cerebral arterioles, as shown in autopsy studies [94,95,168]. It is caused by the mutation of the Notch 3 gene, encoding transmembrane receptor protein, located on chromosome 19 [95]. The mutation results in the fragmentation of the NOTCH3 protein, and the end products are accumulated in the basement membrane and collagen fibers in the cerebrovasculature. Affected patients typically suffer from transient ischemic attack and stroke (found in 85% of symptomatic individuals), migraine with aura (in 80–90% of patients) and progressive cognitive impairment [168,169]. Psychiatric disorders such as apathy and depression are also common. Ischemic episodes are usually lacunar, rarely affect large vessels, and present with pure motor or sensory effects, ataxic hemiparesis, dysarthria and pseudobulbar palsy [170]. Notch 3 gene sequencing is expensive, so the diagnosis usually ensues through skin biopsy: granular osmiophilic material accumulates around vascular smooth muscles, while the endothelium and basal lamina are degenerated [86,171]. Diffuse white matter hyperintensities, especially in the anterior temporal, external capsule and paramedian superior frontal locations, are the usual T2-weighted MRI findings. The disruption of cortical and subcortical networks in the frontal lobe contributes to motor deficits and executive dysfunction [172]. Lacunar strokes and microbleeds are observed with greater prevalence in CADASIL, and unlike the general population, the patients are typically younger than 60 years old and have no typical cardiovascular risk factors. Positron emission tomography, transcranial Doppler sonography and perfusion MRI demonstrate reduced cerebral blood flow, decreased blood volume and impaired metabolism [173]. To date, no disease-modifying treatment is available. As with more common microvascular ischemic diseases, therapeutic suggestions include the strict control of blood pressure, cessation of smoking and statins [174]. Standard treatment for psychiatric disturbances and symptomatic treatment of migraine is recommended.

Among patients with the congenital type of CSVD—cerebral autosomal dominant arteriopathy with subcortical infarcts and leukoencephalopathy (CADASIL)—CMBs are found, according to Lesnik Oberstein et al. in 31% of cases [175]. Other researchers give even greater values. According to Dichgans et al., the prevalence of microbleeding among adult CADASIL patients reaches 69% [143]. It was shown that the most important risk factor for CMB in this group was the age of the patients. However, the degree of disability, the presence of some gene mutations (Notch3-Arg153Cys), the presence of lacunar strokes and the intake of antiplatelet drugs were also important [175]. The risk of microbleeding in people with CADASIL was not associated with the presence of risk factors for cardiovascular diseases [176,177].

## 8. Conclusions

CSVD is a complex group of diseases associated with cerebrovascular architecture disorders. Their pathogenesis is very complex and varies depending on the specific unit. In most cases, they are associated with amyloid angiopathy or arteriosclerosis, but in some contingencies, genetic considerations may also play an important role. As demonstrated in recent years, the manifestation of CVSD can be of great importance in the diagnosis of patients with cognitive impairment. Genetic considerations also play a similar role in vascular diseases such as stroke. A thorough understanding of the role and ethology of cerebral small vessel disease can allow for the more careful monitoring of these groups of patients and the implementation of measures that will prevent relapse or acceleration of the progression of the disease. Developing an accurate knowledge of the meaning and full characteristics of these issues requires further research.

## Figures and Tables

**Table 1:** Cerebral small vessel disease (CVSD) classification. AD—Alzheimer’s disease; CADASIL—cerebral autosomal dominant arteriopathy with subcortical ischemic stroke and leukoencephalopathy; CARASIL—cerebral autosomal recessive arteriopathy with subcortical ischemic and leukoencephalopathy; MELAS—mitochondrial encephalopathy with lactic acidosis and stroke-like episodes; CNS—central nervous system; SLE—systematic lupus erythematosus; HIV—human immunodeficiency virus.

Type:	Description:	Associated Diseases:
Type I	Arteriosclerosis-related CSVD	Hypertension Diabetes
Type II	Amyloid-related CSVD	AD Down’s syndrome
Type III	Genetic CSVD (distinct from amyloid angiopathy)	Fabry’s disease CADASIL CARASIL MELAS Small vessel disease with COL4A1 mutation Retinal vasculopathy with leukodystrophy with TREX1 mutation Hereditary multi-infarct dementia of Swedish type
Type IV	Inflammatory/immunologically mediated CSVD	Systematic Vasculitis: ○ IgA vasculitis ○ Eosinophilic granulomatosis with polyangiitis ○ Granulomatosis with polyangiitis ○ Cryoglobulinemic vasculitis ○ Cutaneous leukocytoclastic ○ Microscopic polyangiitis Primary Central Nervous System Vasculitis Vasculitis secondary to CNS infections tuberculosis, syphilis, HIV, leptospirosis Vasculitis Secondary to Connective Tissue Disorders (SLE, scleroderma, rheumatoid vasculitis, dermatomyositis, Sjogren’s syndrome)
Type V	Venous collagenosis	
Type VI	Other CVSD	Post radiation CVSD Non-amyloid microvessel degeneration in AD

**Table 2:** Comparison of the two most common types of CVSD with clinical and neuroimaging characteristics. CVSD—cerebral small vessel disease, CMBs—cerebral microbleeds.

Classification	Type I	Type II
Characteristic	Associated with arteriosclerosis Related age differences Not associated with amyloid deposition Degenerative microangiopathy	Associated with amyloid deposition Rated age differences
Etiology	Atrophy of smooth muscles in tunica media Aggregate of fibro-hyaline material Stenosis of vessel lumen Degeneration of vessel wall Changing of arterial vessels systems	Vasculopathy Aggregate of amyloid in cortical walls or leptomeningeal small arteries but not capillaries Apolipoprotein gene polymorphism
Manifestations	Lacunar strokes in deep part of brain Dementia and cognitive impairment	Intracerebral hemorrhage in cerebral lobes Non-lacunar strokes Hallmarks of Alzheimer’s disease Cognitive impairment, dementia and transient focal neurological episodes
Radiographic features	CMBs in deep part of the brain Rare changes to the siderosis type of the superficial cortical area Presence of basal ganglia perivascular spaces Hyperintensities in the cerebral region of white matter	CMB sin cerebral lobes Cortical superficial siderosis Centrum seniovate perivascular space Posterior dominance with white matter hyperintensities
